# High tobacco use prevalence with significant regional and sex differences in smokeless tobacco use among Western Alaska Native people: the WATCH study

**DOI:** 10.1080/22423982.2017.1398009

**Published:** 2017-11-12

**Authors:** Kathryn R. Koller, Christie A. Flanagan, Gretchen E. Day, Christi Patten, Jason G. Umans, Melissa A. Austin, Scarlett E. Hopkins, Cheryl Raindl, Bert B. Boyer

**Affiliations:** ^a^ Alaska Native Tribal Health Consortium Division of Community Health Services, Anchorage, AK, USA; ^b^ Mayo Clinic Department of Psychiatry and Psychology, Rochester, MN, USA; ^c^ MedStar Health Research Institute, Hyattsville, MD, USA; ^d^ Georgetown-Howard Universities Center for Clinical and Translational Science, Washington, DC, USA; ^e^ Department of Epidemiology, University of Washington, Seattle, WA, USA; ^f^ University of Alaska Fairbanks Center for Alaska Native Health Research, Fairbanks, AK, USA; ^g^ Texas Biomedical Research Institute, San Antonio, TX, USA

**Keywords:** Cigarettes, smokeless tobacco, Alaska Native, tobacco use patterns

## Abstract

Tobacco use prevalence among Alaska Native (AN) people living in Alaska is greater than the general population prevalence statewide and nationally. Better understanding of regional tobacco use is needed to improve cessation efforts and reduce prevalence. Using self-reported baseline data from the Western Alaska Tribal Collaborative for Health study, we describe tobacco use patterns among AN people in two western Alaska regions. Data were stratified by age group and sex. Dual- and multi-product use in the Yukon-Kuskokwim (Y-K) region was stratified by concurrent vs sequential use. Overall, 87% of the cohort reported having used tobacco. In Norton Sound, cigarette (98%) was the predominant tobacco type. In Y-K 71% smoked, 76% used smokeless tobacco (ST), with 47% reporting use of both products. ST use in Y-K consisted of commercial ST and homemade *iqmik*. Y-K women reported more ST product use, while men reported more cigarette use. Among dual- and multi-product users, the majority reported concurrent use, with no significant differences between men and women. Distinct regional differences include high smoking prevalence in Norton Sound and frequent use of smoking and ST products in Y-K. Findings support modification of cessation programmes to address regional variations in tobacco use patterns.

## Introduction

Since 1991, data collected by the Alaska Behavioural Risk Factor Surveillance System (ABRFSS) [1] have consistently shown greater prevalence of tobacco use among Alaska Native (AN) people over the general Alaska population [2]. In addition, these data [3–6] show greater use of smokeless tobacco (ST) among AN people. Adult prevalence of smoking (36%) [7] and ST use (13%) [8] among AN people, who comprise nearly one-fifth of the state’s population, contributes to the overall statewide prevalence of smoking (22%) and ST use (5%) [9], each exceeding that in the general US population (17% and 4%, respectively) [10,11]. Tobacco cessation programmes have been implemented throughout the state and the Alaska Tribal health systems. However, most cessation programmes and public health messaging in Alaska are geared specifically toward smoking cessation [12] and may not benefit AN people who use ST, either exclusively or along with cigarettes.

Multiple tobacco product use is a growing concern in the US and globally [13–15]. Dual-, poly- and multi-tobacco product use are among the labels ascribed to use of more than one tobacco product, including cigarettes, cigars, pipe, water pipe (hookah), snuff/snus and other tobacco products [13,16,17]. Concurrent use has been variably defined as the use of more than one product within a given day, week or month. Surveillance reports indicate prevalence of multiple tobacco product use is on the rise, especially among young adults [17], while tobacco users who use more than one product report lower tobacco cessation rates [17,18]. Studies with other cohorts, such as adolescents, in which multiple forms of tobacco use are reported, indicate that tobacco cessation interventions and tobacco control policies must address dual and multiple tobacco product use in order to impact tobacco use on a population level [18–20]. Epidemiological studies of tobacco initiation in the US have typically reported product use initiation separately and, among ever users, have not considered initiation of multiple tobacco products sequentially or simultaneously or the type of product used first.

Greater understanding of the patterns of ST use alone and in combination with other tobacco products among AN persons is an essential first step in adapting cessation programmes to address multiple forms of tobacco, especially in communities where ST use is well established. In some Alaska regions, ST use is not only accepted, but considered a healthy alternative to smoking and closely tied to cultural lifestyle [6,21,22]. Examining regional patterns of tobacco use can reveal population-based information pertinent to public health messaging that is accurate, consistent and culturally appropriate.

We sought to describe tobacco use patterns among male and female AN people in two rural western Alaska regions, Norton Sound and Yukon-Kuskokwim (Y-K) Delta, where tobacco use prevalence exceeds that of both the Alaska and US general populations. The 13 communities along the coast of the Norton Sound region are located on the Seward Peninsula in northwestern Alaska [23]. Census data for 2010 show that ~ 80% of Norton Sound residents are AN heritage [24]. While comparable in geographic size, Norton Sound’s population is less than 1% of that of West Virginia [25]. The Y-K region encompasses an area more than triple that of Norton Sound. It is situated immediately south of Norton Sound, with more than 50 rural communities along the coast and banks of the Yukon and Kuskokwim Rivers [26]. About 90% of Y-K residents are AN heritage [24]. The Y-K region is larger than the state of Washington, which, by comparison, has a population of more than 300 times that of the Y-K region [27].

While survey data from the ABRFSS demonstrate differences in smoking and ST use in Norton Sound and Y-K, survey data collected during the study timeframe did not differentiate between commercial ST and *iqmik* [28], a homemade form of ST that is embedded in cultural practices [4], nor do they report on the use of more than one tobacco product and whether or not product use was concurrent or sequential. For our analysis, a special focus on the use of ST included *iqmik*. Among dual users for whom data were available, we also assessed first product used and distinguished between simultaneous or sequential initiation.

## Methods

In 2009, investigators with the Western Alaska Tribal Collaborative for Health (WATCH) began consolidating data from multiple Alaska Native cohort studies, as described previously [29]. Briefly, during 2000–2004, three population-based cohort studies were conducted in two adjacent western Alaska regions: the Genetics of Coronary Artery Disease in Alaska Natives (GOCADAN) study [30], based in the Norton Sound region, and the Centers for Alaska Native Health Research (CANHR) [31] and Education and Research Towards Health (EARTH) [32] studies, based in the Y-K Delta region. Each study enrolled large proportions of adults in each rural community involved, resulting in samples with characteristics reflecting the regional adult population. However, individual cohorts were small, limiting statistical power and reliability. The considerable overlap in tobacco use information collected at baseline by each cohort fostered data consolidation, thus providing a unique opportunity to characterise tobacco use prevalence and patterns of use in these two western Alaska regions. Approval to consolidate the study data was granted by the institutional review boards for the Alaska Area, MedStar Health Research Institute and the University of Alaska Fairbanks. Tribal approval was received from the Alaska Native Tribal Health Consortium, Norton Sound Health Corporation and Yukon-Kuskokwim Health Corporation.

The CANHR and GOCADAN studies collected self-reported demographic and tobacco use data by in-person interviews with trained study personnel. The EARTH study collected similar self-reported data using audio computer-assisted self-interview methods [33,34]. Demographic data obtained by all studies included age, sex and education level in years of school completed. The WATCH study cohort included only Alaska Native participants; specifically, Inupiat AN in Norton Sound and Yup’ik/Cup’ik AN in Y-K. Between the EARTH and CANHR studies, enrolment was conducted in more than half of the Y-K communities, including Bethel, the regional hub. For GOCADAN, enrolment occurred in eight of the 13 Norton Sound communities and included the hub community of Nome.

Participants in each study were asked to specify tobacco types used, specifically cigarettes and ST (chew or snuff), age at initiation and age at quitting (if relevant). In each study, participants were asked if they had ever smoked ≥ 100 cigarettes in their lifetime. Those who reported “yes” to the question were considered ever smokers. We defined current smokers as those who reported ever smoking and did not report an age for quitting or whose reported age at quitting was within 1 year of their age at the baseline examination. Alternatively, age at quitting was derived from reported age of smoking initiation and reported years smoked. Former smokers included participants whose age at baseline was at least 1 year older than the reported (or derived) age at which they quit. We defined ever use of ST as self-reported use of any commercial or homemade ST products. These data enabled us to determine baseline prevalence of non-tobacco use, former and current cigarette smoking and ever using ST in both study regions. We were also able to determine prevalence of exclusive ever smoking, exclusive ever ST use and ever use of both cigarettes and ST. Users of both products were defined as ever having used cigarettes and having ever used ST.

For ST use, the CANHR and EARTH studies, both conducted in the Y-K Delta, specifically asked participants to specify whether product use included commercial ST and/or *iqmik*, a homemade ST product commonly used in the Y-K region [4,6,21,22,35,36]. We analysed *iqmik* use separately from commercial ST, due to inherent differences in product content and cultural beliefs and acceptance. While commercial ST and *iqmik* both contain tobacco, *iqmik* contains ground ash produced by burned *Phellius iganiarius* or “punk”, a tree fungus [4]. The ash raises the pH, which promotes nicotine absorption by increasing the percentage of unionised nicotine bioavailable in the product. Previous exploratory studies conducted in western Alaska reported that, although tobacco has no ceremonial use, *iqmik* is considered a “natural” product and, thus, its use is considered a healthier practice than cigarette smoking. Hence, *iqmik* use is culturally acceptable for women to use during pregnancy and, in some communities, for young children to assist in its production with pre-mastication prior to storage and later adult consumption [37,38].

For each tobacco type selected, the CANHR and EARTH studies additionally asked participants to provide the age they began using and either the number of years they used or the age at which they quit using the products, if applicable. Thus, in the Y-K region only, we were able to determine prevalence of ever using two tobacco products (dual-use) and ever using more than two tobacco products (multi-use) as well as whether this use was concurrent or sequential. Ever dual-use was defined as use of any combination of two tobacco products (cigarettes and commercial ST, cigarettes and *iqmik*; or commercial ST and *iqmik*) and ever multi-use was defined as use of all three products during one’s lifetime. Sequential use was defined as reporting cessation of one tobacco product in 1 year and the uptake of a new product in either the same year or in later years. Concurrent use was defined as any overlap in the years of use of two or more products, even if the participant reported cessation of all but one product subsequent to the overlap. Importantly, we were able to determine which tobacco product was used first (cigarettes, commercial ST or *iqmik*) or if these products were initiated simultaneously. Participants reporting they had initiated any tobacco use with more than one product in the same year were considered simultaneous starters.

The WATCH Operations Committee was responsible for data consolidation. Members identified similar variables for all baseline data collected in the original studies [29]. Data collected identically (e.g. clinical and anthropometric measurements) were combined. Data collected similarly, but not identically (such as tobacco use), were harmonised into newly defined variables. Variables too dissimilar by collection method or definition were not incorporated into the dataset.

Using SAS version 9.4 (Cary, NC), we calculated means with standard deviations for continuous variables (age and years of education) and frequencies with percentages for categorical data, stratifying by region and sex. Differences in frequencies between strata were assessed using Chi-square statistic for categorical data and Student *t*-test for continuous data. We assessed differences between Y-K participants who were concurrent cigarette and ST users (commercial ST or *iqmik*) and participants who were exclusive smokers and exclusive ST users using logistic regression analysis, adjusting for age, sex and education. Significance was determined at p<0.05.

## Results

### Tobacco use among WATCH participants

Table 1 provides baseline characteristics for the entire sample of 3,932 WATCH participants, highlighting differences between the 67% residing in the Y-K region and those from the Norton Sound region. While most (87%) participants reported having ever used tobacco, Table 1 shows that nearly all Norton Sound participants had used tobacco, essentially all were smokers, with three-quarters being current smokers and few having used ST. In contrast, while ever use of tobacco was higher in the Y-K region, most had used ST, even more so among women.

**Table 1.** Baseline characteristics and tobacco use among Alaska Native adults living in Norton Sound and Yukon-Kuskokwim delta regions.

Baseline characteristics/tobacco use	Norton Sound	Yukon- Kuskokwim	*p*-value (region)	Total WATCH	p-value (sex)
Sample size, n (%)					
Total	1,318 (33)	2,614 (67)	<0.001	3,932 (100)	
Men	596 (45)	1,230 (47)	0.276	1,826 (46)	<0.001
Women	722 (55)	1,384 (53)		2,106 (54)	
Age, median years (range)					
Overall	41 (18–95)	37 (18–94)	<0.001	38 (18–95)	
Men	40 (18–88)	36 (18–86)	<0.001	37 (18–88)	<0.001
Women	42 (18–95)	38 (18–94)	0.015	39 (18–95)	
Education, mean years (SD)*					
Overall	11.7 (±2.3)	9.8 (±3.1)	<0.001	10.6 (±2.9)	
Men	11.8 (±2.0)	9.9 (±3.0)	<0.001	10.7 (±2.8)	0.134
Women	11.6 (±2.6)	9.7 (±3.2)	<0.001	10.5 (±3.1)	
Ever used tobacco, n (% sample)					
Overall	1,084 (82)	2,330 (89)	<0.001	3,414 (87)	
Men	510 (86)	1,134 (92)	<0.001	1,644 (90)	<0.001
Women	574 (80)	1,196 (86)	<0.001	1,770 (84)	
Ever smoked, *n* (% ever used tobacco)					
Overall	1,058 (98)	1,646 (71)	<0.001	2,704 (79)	
Men	489 (96)	965 (85)	0.074	1,454 (88)	<0.001
Women	569 (99)	681 (57)	<0.001	1,250 (71)	
Current smokers, n (% ever smoked)					
Overall	801 (76)	833 (51)	<0.001	1,634 (60)	
Men	377 (77)	530 (55)	<0.001	907 (62)	0.025
Women	424 (75)	303 (45)	<0.001	727 (58)	
Former smokers, *n* (% ever smoked)					
Overall	257 (24)	813 (49)	<0.001	1070 (40)	
Men	112 (23)	435 (45)	<0.001	547 (38)	0.025
Women	145 (26)	378 (56)	<0.001	523 (42)	
Ever used ST, n (% ever used tobacco)					
Overall	93 (9)	1,777 (76)	<0.001	1,870 (55)	
Men	72 (14)	778 (69)	<0.001	850 (52)	0.238
Women	21 (4)	999 (84)	<0.001	1020 (58)	
Ever smoked and used ST, n (% ever used tobacco)					
Overall	67 (6)	1093 (47)	<0.001	1160 (34)	
Men	51 (10)	609 (54)	<0.001	660 (40)	<0.001
Women	16 (3)	484 (41)	<0.001	500 (28)	

n, number; SD, standard deviation; ST, smokeless tobacco; smoked or smokers=cigarette users; significance at p<0.05.

*Years of education were obtained from 2,717 of 3,932 (69%) of total sample reporting tobacco use status; 1,152 of 1,318 (87%) in Norton Sound and 1,565 of 2,614 (60%) in Yukon-Kuskokwim.

Among those who ever used tobacco, results are broken down further for ever smokers, ever ST users and ever dual users (smoked and used ST). Among ever smokers, results are broken down further for current and former smokers.

### Dual and multiple product use (Y-K region only)

**Figure 1.**
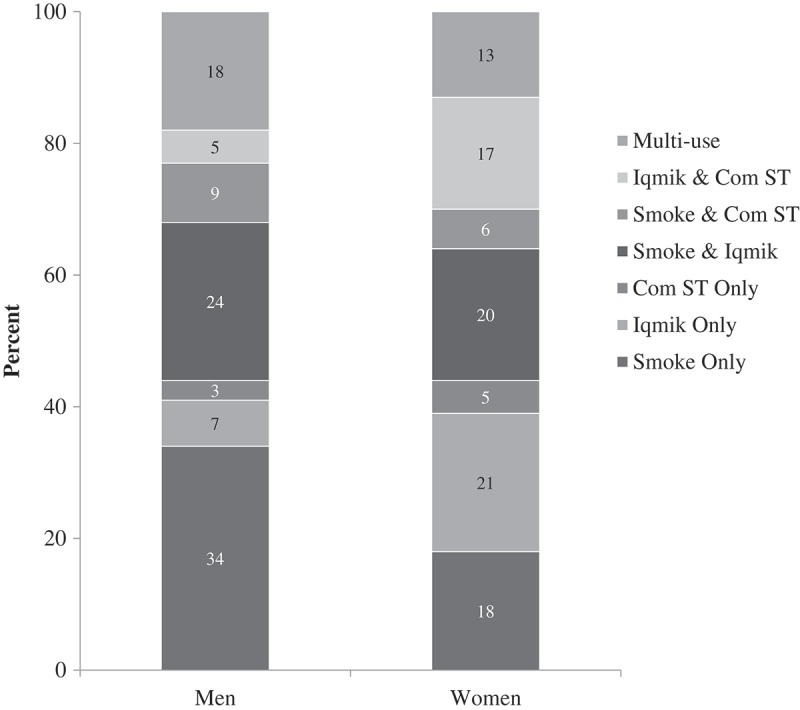
Differences in tobacco types used by 1,081 men and 1,157 women in the Yukon-Kuskokwim region. *Note*: Total n=2,238 ever tobacco users (96% of Y-K sample reporting ever tobacco use).

We analysed additional data on single, dual- and multi-product use from 96% of the 2,330 ever tobacco users in the Y-K region. Of these, 43% (964/2238) reported single-product use, the remainder reporting ever use of two or more tobacco products (41% dual-use and 16% multi-product use), with substantial differences in tobacco type observed between men and women (Figure 1). Among those who used only one form of tobacco, men most commonly used cigarettes, while women most commonly used *iqmik*. Very few ever tobacco users reported single use of commercial ST. Dual-use was more prevalent among women (p=0.04). While proportionately more men than women ever tobacco users reported dual use of cigarettes and either commercial ST or *iqmik* (each p<0.001), more women than men ever tobacco users reported having used commercial ST and *iqmik* (p<0.001). Prevalence of multi-product use (ever use of cigarettes, commercial ST and *iqmik*) was more common among men than women (p=0.004).

### First tobacco product use (Y-K region only)

The original study conducted in Norton Sound did not assess differences in ST product use (commercial vs *iqmik*). However, very few Norton Sound residents report ST use, while ST, specifically *iqmik*, is far more common to the Y-K region. Therefore, only Y-K data were available to analyse when participants started and ended use of each product. Participants in each study were also allowed to skip questions they were not comfortable answering. Despite these limitations, a sub-set of 701 of 920 (76%) Y-K ever dual-users and 270 of 354 (76%) Y-K multi-product users provided information on which tobacco product type(s) they had used first (Table 2). The sub-set consisted of 294 (42%) Y-K men and 407 (58%) Y-K women. Dual-use was grouped by ever use of cigarettes and commercial ST, ever use of cigarettes and *iqmik* and ever use of commercial ST and *iqmik*. We calculated percentages of each group separately to illustrate the group and sex differences. Among dual-users of cigarettes and ST, whether *iqmik* (n=303) or commercial ST (n=150) was used, similar majorities initiated tobacco use with ST, although with higher proportions among women. Among dual-users who did not smoke (n=248), commercial ST was the product first used by 51% of the group, while 43% reported initiating both ST products simultaneously. Simultaneous starters comprised the largest proportion (39%) of multi-product users.

**Table 2.** Type tobacco first used by Alaska Native dual- and multi-product tobacco users in the Yukon-Kuskokwim (Y-K) region.

	First product used in 701 Y-K dual- & multi-product users				
Product used	Sample, n	Cigs, n (%)	Com ST, n (%)	Iqmik, n (%)	Simultaneous starters, n (%)
*Dual-product use*					
Cigarettes and Iq’mik					
Overall	303	76 (25)	—	192 (63)	35 (12)
Men	151	46 (31)	—	85 (56)	20 (13)
Women	152	30 (20)	—	107 (70)	15 (10)
Cigarettes and commercial ST					
Overall	150	40 (27)	92 (61)	—	18 (12)
Men	90	31 (34)	51 (57)	—	8 (9)
Women	60	9 (15)	41 (68)	—	10 (17)
Commercial ST and Iqmik					
Overall	248	—	127 (51)	15 (6)	106 (43)
Men	53	—	31 (59)	3 (6)	19 (36)
Women	195	—	96 (49)	12 (6)	87 (45)
*Multi-product use*					
Cigarettes, commercial ST and Iq’mik					
Overall	270	60 (22)	74 (27)	31 (12)	105 (39)
Men	154	39 (25)	40 (26)	15 (10)	60 (39)
Women	116	21 (18)	34 (29)	16 (14)	45 (39)

%=number (overall, men and women) identifying the first tobacco product used divided by the total (overall, men and women) in that product use group.

Post-initiation, most dual-use (61%) and multi-use (73%) was concurrent among ever tobacco users, which did not differ by sex (Table 3). Compared to exclusive ST use (either commercial ST or *iqmik*), women were less likely than men (OR=0.26) to use cigarettes and ST concurrently (Table 4). When compared instead to exclusive cigarette use, women were more likely than men (OR=1.5) to be concurrent cigarette and ST users. Concurrent cigarette and ST users were also younger.

**Table 3.** Concurrent vs sequential use by Alaska Native dual- and multi-product tobacco users in the Yukon-Kuskokwim (Y-K) region.

	Concurrent vs sequential use in 993 Y-K dual- & multi-product users			
Use group	Sample, n	Sequential use, n (%)	Concurrent use, n (%)	p-value (sex)
Dual-product use				
Overall	722	281 (39)	441 (61)	
Men	397	149 (38)	248 (63)	0.398
Women	325	132 (41)	193 (59)	
Multi-product use				
Overall	271	73 (27)	198 (73)	
Men	155	42 (27)	113 (73)	0.946
Women	116	31 (27)	85 (73)	

%=number (overall, men and women) identifying the first tobacco product used divided by the total (overall, men and women) in that product use group. This table examines men and women who were dual and multi-tobacco product users and whether their use of more than one product was sequential or concurrent.

**Table 4.** Associations between socio-demographic factors and concurrent cigarette and ST use among adults in the Yukon-Kuskokwim (Y-K) region.

Factor	OR (95% CI)	p-value
*Concurrent cigarette and ST use compared to exclusive ST use*		
Model 1 (n=1,065)		
Sex (Referent: Male)	0.26 (0.20–0.35)	<0.001
Age (Linear)	0.96 (0.95–0.97)	<0.001
*Concurrent cigarette and ST use compared to exclusive cigarette use*		
Model 2 (n=934)		
Sex (Referent: Male)	1.49 (1.14–1.95)	<0.001
Age (Linear)	0.98 (0.97–0.99)	0.004

OR, odds ratio; CI, confidence interval; significance = p-value<0.05. This table provides a comparison between dual and mono tobacco product users. The results show that women are far less likely than men (0.26; p<0.001) to use two products (cigarettes and ST) as compared to just ST. However, when comparing dual use to just cigarette smoking, women are more likely than men (1.49; p<0.001) to use both cigarettes and ST.

## Discussion

This study characterises tobacco use prevalence and patterns of use among AN people in the Norton Sound and Y-K regions, with significant regional and sex differences observed in ST use. Tobacco use in the Norton Sound region was limited almost exclusively to smoking, for both men and women. In the Y-K region, men were more likely to have been smokers, while women were more likely to have used ST. Importantly, half of Y-K participants who reported having used tobacco also reported dual- or multi-product use.

Y-K women who used tobacco were significantly more likely to be dual-users compared to men who used tobacco. Among dual-users, women were more likely to have used commercial ST and *iqmik*, whereas men were more likely to have used cigarettes and commercial ST or cigarettes and *iqmik*. Male tobacco users were significantly more likely to be multi-product users of cigarettes, commercial ST and *iqmik* compared to female tobacco users. Among dual-users of cigarettes and ST (i.e. commercial ST or *iqmik*), more than 60% reported tobacco initiation with ST. For dual-users who used commercial ST and *iqmik*, more than 40% were simultaneous starters of both ST products. Furthermore, there was a high prevalence of concurrent tobacco product use among Y-K participants.

We recognise data collection for this project are dated, collected between 13 and 17 years ago, and that tobacco use and prevalence has since changed statewide and nationally [39]. However, data suggests that smoking prevalence has not significantly decreased among AN people from 1996 (47%) to 2014 (42%), compared to non-Native Alaskans who have experienced a significant decrease (1996, 25%; 2014, 17%) [39]. For ST use the prevalence has remained relatively stable between 1996 and 2014 for AN people (12% and 15%, respectively) and non-Native Alaskans (4% both years). These percentages illustrate that tobacco use prevalence remains disproportionately high among AN people in comparison to non-Native Alaskans.

When stratified by region, data from the ABRFSS reports lower percentages of tobacco use for both cigarettes and ST than what our results demonstrate [28]. However, ABRFSS data is aggregated over several years to achieve sufficient power due to small sample sizes. For this project, three cohorts were combined, which increased power, reducing the probability of a type II error. Furthermore, population-based data were obtained and assessed in-person rather than by phone survey. Regional Tribal leaders determined which communities were to be involved in the research. Due to small population sizes, we do not name specific communities. When compared to census data, baseline characteristics other than sex, with more women represented in our study, were similar [32,40,41]. The inclusion of most eligible adult residents in more than half of the rural remote communities in each region provides a more realistic representation of tobacco use patterns across the two regions than phone survey data aggregated over multiple years.

Harmonised data have been consolidated and redefined to incorporate a larger dataset. Harmonisation, thus, results in data that are less precise than originally collected. While the WATCH tobacco data may be less precise than in their original form, they are more precise than any data collected by phone surveys conducted nationally or regionally in this population. Our findings affirm ABRFSS reports that cigarette use in Norton Sound and ST use in Y-K are the most prevalent types of tobacco used by AN adults in those regions [7–9]. Other research has found that female AN youth were more likely to use ST compared to male youth in Y-K [21]. Additionally, research conducted with AN people living in the Bristol Bay region, which borders the Y-K to the south, found women more likely to use *iqmik* compared to men [42]. Availability, tradition, and cultural beliefs may contribute to the differences we observed in *iqmik* use between regions. *Iqmik* has been used in the Y-K region for more than 150 years and is perceived to be a natural, thus safer, alternative to cigarette smoking [35]. Fungus that grows on birch trees, which are more available in the Y-K and Bristol Bay areas than the Norton Sound region, is a key ingredient used to make *iqmik*. This availability may also contribute to some difference in use observed between the two regions. Patterns of ST use among AN women are unlike those reported for women nationally and our results support findings for high prevalence of *iqmik* use by AN women reported in other studies [38,42]. The use of ST among girls and women in other countries has emerged as an important public health concern [43,44].

Only one other study assessed dual-product use by AN people, noting that men were more likely than women to be dual users [42]. That study focused only on current tobacco users in the Bristol Bay region, and did not differentiate between commercial ST and *iqmik*. Recent US studies demonstrate that dual- or multi-product use is most prevalent among young adults [16,20,45]. While these studies report up to 30% of young adults are dual product users [19], this percentage is far lower than in AN residents of the Y-K region. Among Native American adults in North Carolina, one study found only 5% of current tobacco users were dual users of cigarettes and ST [46]. While we assessed dual-and multi-product use among ever tobacco users (vs current users only), dual- or multi-product tobacco use appears to be far greater among AN persons than in other populations.

The majority of adult dual commercial ST and *iqmik* users as well as multi-product users in the Y-K region reported initiating more than one tobacco product within the same year. However, dual users of cigarettes and ST were more likely to have initiated tobacco with a ST product, consistent with a previous retrospective review of medical record review from 665 AN adolescents in the Y-K region documenting that tobacco use was most commonly initiated with ST [21].

This study is also the first to explore sequential and concurrent tobacco use among AN adults reporting having used more than one tobacco product. Use of cigarettes after initiating ST is also seen among snus users in Norway [47]. Nationally, there is increasing concern over concurrent dual- and multi-product tobacco use. Research using Minnesota statewide data found the proportion of cigarettes smokers reporting ST use increased between 2007 and 2010 [18]. It has been suggested that the increase in ST use among smokers may be due to wide implementation of in-home and public smoking bans nationwide and the tobacco industry marketing of ST as a substitute for cigarettes when smoking is not permitted [48]. However, many of our study data pre-date these more recent trends in policy and marketing, with many Y-K smokers reporting concurrent use well before public bans.

The strengths of our study are the large sample size of AN persons, data on multiple tobacco products (cigarettes, along with separate assessment of commercial ST and *iqmik*) and use patterns (concurrent or sequential) and longstanding relationships with the respective communities. Nicotine and carcinogen bioavailability appear to vary dramatically across these tobacco products [49]. This study is limited to assessment of cigarettes, commercial ST and *iqmik*, mainly due to the minimal use of pipe or cigar smoking in these regions and by the collection of these baseline data well before the widespread use of e-cigarettes. Further, data on ST use patterns were limited to the Y-K Delta region, since cigarette use predominated in the Norton Sound region.

Additional research is needed to explore *iqmik* content, potential harmful health effects of *iqmik* use and the perceptions of its use, particularly in the Y-K region. Among Y-K women, tobacco use is driven primarily by *iqmik* use. This is explained by the lower likelihood of women being dual cigarette and ST users than exclusive ST users and the higher likelihood of women being dual cigarette and ST users when compared to exclusive smokers. Research has demonstrated that dual- and multi-product tobacco users experience lower cessation rates [17,18], possibly due to greater cotinine and dependence levels [50]. On average, AN smokers living in Bristol Bay smoke about half as many cigarettes (7.8) per day [42] as US smokers (14.2) [51], but demonstrate similar plasma cotinine levels (170 ng/mL and 200 ng/mL, respectively), suggesting altered nicotine metabolism [49,52]. These changes in nicotine pharmacokinetics imply the need for a increased dosing of nicotine replacement therapy to aid in smoking cessation, a need that may extend to other AN populations, such as those in the Y-K or Norton Sound regions.

Clearly, tobacco prevention efforts, cessation programmes and public health messaging must resonate with each target population. Our study demonstrates wide variations in tobacco product use between two Alaska regions and between men and women within the Y-K region. These findings emphasise the need for tailoring prevention and cessation interventions to meet regional and community needs based on tobacco product use. Given our results, we recommend that, in Norton Sound, tobacco interventions focus on cigarette cessation and prevention for both males and females. In the Y-K region, cessation efforts should be directed towards reducing ST use and dual-product tobacco use for both men and women. Programmes should have information on the benefits of stopping smoking, commercial ST and *iqmik* use and be prepared to address both mono and dual-product use. Prevention interventions should emphasise the harms of dual- and multi-product tobacco use.
